# Template Removal Strategies in Electropolymerized Molecularly Imprinted Polymers: Mechanisms, Challenges, and Perspectives

**DOI:** 10.3390/s26123742

**Published:** 2026-06-12

**Authors:** Julio Ojeda, Angie Castillo-Barzola, Sthefanny Pamela Arauco Bendezú, José Luiz da Silva, Karin Chumbimuni-Torres

**Affiliations:** 1 Department of Chemistry, University of Central Florida, Orlando, FL 32816, USA; juliohector.ojedavelarde@ucf.edu (J.O.); angie.castillobarzola@ucf.edu (A.C.-B.); 2 Laboratorio de Electroquímica, Electroanalítica y Materiales Aplicados, Laboratorios de Investigación y Desarrollo, Facultad de Ciencias e Ingeniería, Universidad Peruana Cayetano Heredia, San Martín de Porres, Lima 150135, Peru; sthefanny.arauco.b@upch.pe (S.P.A.B.); jose.da.silva@upch.pe (J.L.d.S.)

**Keywords:** template removal, molecularly imprinted polymers, electropolymerization

## Abstract

**Highlights:**

**What are the main findings?**

- Template removal is a critical factor affecting recognition-site accessibility, polymer integrity, and sensor performance in e-MIPs.
- PPy- and PoPD-based e-MIPs exhibit different responses to removal conditions due to their distinct chemical and electrochemical properties.

**What are the implications of the main findings?**

- Polymer-specific removal strategies are needed to improve reproducibility, selectivity, and analytical reliability in e-MIP sensors.
- Standardized reporting of removal conditions is essential to enable comparison across studies and support practical sensor development.

**Abstract:**

Template removal represents a critical yet often underexplored step in the fabrication of electropolymerized molecularly imprinted polymers (e-MIPs), directly influencing cavity integrity, selectivity, and sensor performance. In this review, we provide a comprehensive analysis of the most commonly employed template removal strategies, including immersion-based methods and electrochemical cleaning, with a particular focus on systems based on polypyrrole (PPy) and poly(o-phenylenediamine) (PoPD). We examine how template removal conditions, such as solvent composition, pH, and applied potential, affect polymer structure, doping state, swelling behavior, and electrochemical properties. Special attention is given to mechanistic aspects such as protonation/deprotonation, overoxidation, and polymer–template interactions, which govern both remotion efficiency and potential degradation pathways. By comparing PPy and PoPD systems, we highlight how intrinsic polymer properties dictate the suitability of specific removal strategies. Additionally, we discuss emerging approaches, including multi-step template removal protocols and the incorporation of conductive nanomaterials to mitigate performance loss. This work aims to provide a mechanistic perspective on how template removal conditions affect polymer structure, electrochemical properties, and the overall performance of e-MIP-based sensors.

## 1. Introduction

Electrochemical sensors have emerged as promising analytical platforms due to their portability, low cost, rapid response, and compatibility with miniaturized devices. However, their performance strongly depends on the selectivity of the recognition layer. Molecularly imprinted polymers (MIPs), particularly electropolymerized MIPs (e-MIPs), have attracted considerable attention as synthetic recognition materials capable of selectively recognizing a wide range of targets, including drugs, biomarkers, pesticides, hormones, proteins, and environmental contaminants. Their robustness, chemical stability, and compatibility with electrochemical transduction make them attractive alternatives to biological receptors in sensing applications [1,2]. Conventional analytical techniques, such as high-performance liquid chromatography (HPLC) coupled with ultraviolet/visible (UV/Vis) or mass spectrometry detection, are widely regarded as reference methods due to their sensitivity and reliability. However, the high cost, operational complexity, and maintenance requirements of such instrumentation limit their accessibility, particularly in resource-constrained settings. These limitations highlight the need for alternative technologies that are affordable, robust, and easy to operate.

Chemical sensors offer a promising route toward solving that limitation. They typically require minimal sample preparation, deliver easily interpretable readouts, and can be deployed directly in the field. Among them, electrochemical sensors stand out for their simplicity, portability, and versatility, attributes exemplified by commercial glucose and pH sensors. To achieve selective detection of a given analyte, these sensors must incorporate recognition materials capable of distinguishing the target molecule from a complex background. Moreover, these materials must be chemically stable and exhibit long shelf lives to ensure consistent performance.

**Figure 1:**
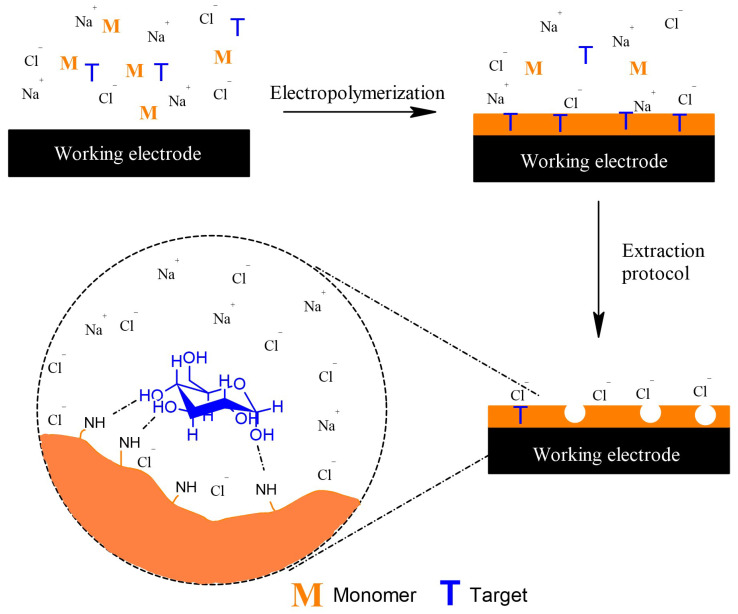
Schematic representation of e-MIP electrosynthesis.

Molecularly imprinted polymers (MIPs) represent an attractive class of synthetic recognition elements that meet these criteria. MIPs contain molecular cavities complementary in size, shape, and functional groups to their target analytes. They are typically prepared by polymerizing functional monomers in the presence of a template molecule, followed by removal of the template to leave imprinted binding sites (Figure 1). Owing to their robustness and reusability, MIPs have been successfully applied to detect a wide variety of targets, often without the need for matrix pretreatment.

MIPs can be synthesized through several routes, including precipitation, core–shell, and electropolymerization methods. The first two techniques generally yield powdered “bulk-MIPs,” whereas electropolymerization produces thin, surface-confined films. In these cases, polymerization is initiated by the addition of a radical initiator. In the case of electropolymerization, polymer growth is triggered by the application of a specific voltage that generates the reactive monomer radicals. Parameters such as applied voltage, number of cycles, and scan rate also influence radical formation and thereby enable fine control over film thickness and morphology, an advantage over conventional chemical polymerization methods.

A critical step, yet often underestimated step in MIP fabrication, is the removal of the template molecule from the polymer matrix [3,4]. If the template removal procedure is too harsh, it can damage the functional groups that define the cavity’s selectivity, resulting in loss of binding capability [5]. This is often evident when both MIP and its non-imprinted polymer (NIP, a target-free MIP) exhibit similar adsorption capacity or when the imprinted sites undergo “template leaching”, losing their ability to retain the analyte. Conversely, if template removal is too mild, residual template molecules may remain trapped within the polymer matrix, blocking access to recognition sites. Therefore, careful optimization of removal conditions is essential to achieve complete template extraction without compromising cavity integrity.

Although several reviews discussing molecularly imprinted polymers, electropolymerized MIPs, and template removal strategies have previously been published [2,6,7], these works mainly provide broad overviews of fabrication protocols and applications. In contrast, the mechanistic relationship between washing conditions and the physicochemical nature of electropolymerized polymer matrices remains insufficiently discussed. To address this gap, this review critically examines how template removal conditions affect polymer properties, with particular emphasis on the structural and functional changes induced in the polymer network during extraction.

To provide a focused and mechanistically consistent discussion, we restrict our analysis to e-MIPs synthesized from pyrrole (Py) and o-phenylenediamine (oPD), two of the most widely employed monomers in electrochemical MIP sensors [8,9]. Although other monomers, such as aniline- and thiophene-based systems, are also relevant in the field, limiting the scope to PPy and PoPD allows a more detailed analysis of how extraction conditions influence conductivity, doping behavior, overoxidation, swelling, and cavity integrity. Insights obtained from these systems can nevertheless be extended to other electroactive polymers. Ultimately, this review aims not only to summarize current template removal protocols but also to critically discuss their advantages, limitations, and impact on polymer performance. By deepening the understanding of this often-overlooked fabrication step, we seek to improve the reproducibility, reliability, and applicability of e-MIP sensors.

## 2. Theoretical Background

The selectivity of MIPs arises from the specific intermolecular interactions formed between the template molecule and the functional groups of the polymer matrix (Figure 1). These interactions typically include hydrogen bonding, π–π stacking, dipole–dipole forces, or a combination. The strength and geometry of these interactions are often evaluated before polymerization by studying the monomer–template complex, using techniques such as proton nuclear magnetic resonance (^1^H NMR) titration or computational modeling [10,11,12]. Numerous studies have demonstrated that this preliminary characterization provides a reliable estimate of the binding performance of the resulting MIP, whether synthesized in bulk or via electropolymerization (e-MIP) [10].

Preserving these functional moieties during template removal is critical for achieving both sensitivity and selectivity. Inappropriate removal conditions can chemically alter the recognition sites through side reactions such as oxidation, hydrolysis, or unintended doping of the polymer [13]. Furthermore, certain removal media can disrupt the polymer network itself [14]. Although such disruption may not directly modify the functional groups within the cavity, it can compromise the film’s structural integrity and hinder signal transduction. For instance, polymers tend to swell in solvents [15,16,17] and this swelling can influence the diffusion of the template and the accessibility of the imprinted cavities. In e-MIPs, excessive swelling and/or partial delamination of the thin polymer layer may impair electron transfer and degrade analytical performance.

A notable difference between e-MIPs and bulk MIPs is the presence of cross-linkers in the latter. Cross-linkers reinforce the polymer framework, enhancing its mechanical and chemical stability and enabling the swelling–shrinking process to remain partially reversible [18,19,20]. In contrast, e-MIPs often rely on self-limiting films where the layer thickness is controlled by the applied charge rather than by external cross-linkers, which results in less bulk-rigid layers that are strongly coupled to the electrode surface [21,22].

### 2.1. The Nature of Pyrrole-Based Polymers

**Figure 2:**
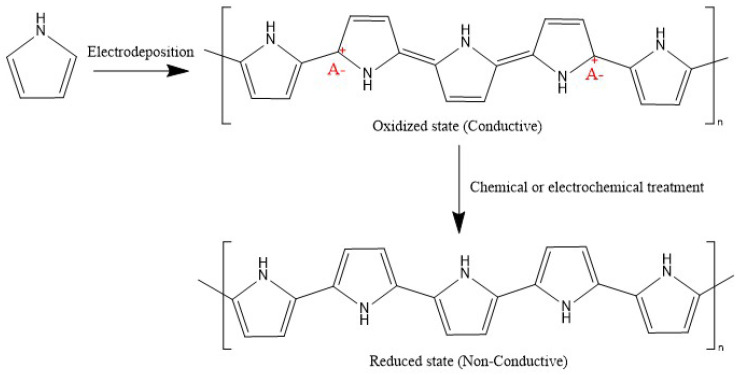
Polypyrrole is formed as a conductive polymer after electrodeposition, but it can be changed to its reduced state via chemical or electrochemical treatment. A^−^: anion.

Polypyrrole (PPy) is a conjugated polymer composed of repeating Py units, five-membered heteroaromatic rings containing one nitrogen atom. Although ideally linear, PPy can exhibit diverse structural motifs, including branched or cross-linked segments and chain regions of varying conjugation lengths [23]. One of the most common synthetic routes is the electropolymerization of pyrrole in aqueous media (Figure 2). During electropolymerization pyrrole monomers are oxidized to form radical cations that couple predominantly through their α–α positions, yielding PPy in its oxidized, conductive state [24]. To maintain electroneutrality, anions from the electrolyte are incorporated into the growing polymer matrix as dopants. The resulting material possesses a positively charged backbone balanced by counteranions, species that strongly influence the polymer’s morphology, electrochemical behavior, and stability [25].

A wide range of anions can act as dopants, including inorganic species such as perchlorate (ClO_4_^−^), nitrate (NO_3_^−^), sulfate (SO_4_^2−^), chloride (Cl^−^), and bromide (Br^−^), as well as organic anions such as p-toluenesulfonate (pTS^−^) and dodecylbenzenesulfonate (DBS^−^) [24,25]. The choice of dopant critically affects film compactness, ion transport, and mechanical flexibility. A key advantage of employing conductive polymers such as PPy in e-MIP sensors is that, when the target analyte is electroactive, it can be detected directly through voltammetric techniques such as cyclic or square-wave voltammetry.

PPy can also exist in a reduced, non-conductive form, obtained either by electrochemical reduction or by alkaline treatment. Under strongly oxidative or basic conditions, PPy undergoes overoxidation, leading to the expulsion of dopant anions and the formation of an insulating, oxygen-functionalized network. This overoxidized PPy behaves as a charge and size-exclusion membrane, selectively permitting cation transport while hindering anion diffusion [26]. In such systems, detection of non-electroactive targets is typically achieved indirectly by monitoring the response of an external redox probe, most commonly the ferri/ferrocyanide redox couple, before and after template binding.

That is the reason why template removal can influence not only the removal of the template molecule but also the oxidation state and thus the conductivity of the PPy matrix. Because the removal conditions may shift the polymer between its reduced and oxidized forms, these effects cannot be completely eliminated. However, they can be controlled by subjecting the NIP to identical removal conditions. Despite these intrinsic limitations, PPy remains one of the most attractive materials for e-MIP fabrication owing to its high electrical conductivity, environmental stability, biocompatibility, and ease of synthesis.

### 2.2. The Nature of o-Phenylenediamine Based Polymers

**Figure 3:**
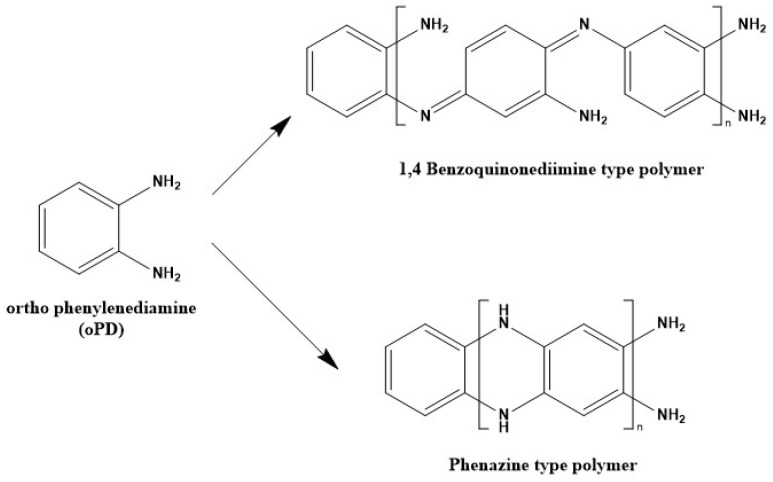
Poly-oPD formation as a benzoquinonediimine type or phenazine type.

Poly(o-phenylenediamine) (PoPD), obtained from the electropolymerization of the o-phenylenediamine (oPD) monomer (Figure 3), contains two amine groups (–NH_2_) that play a central role in the monomer–template interactions. For e-MIP fabrication, oPD is typically dissolved in aqueous buffer solutions at low pH to improve solubility, since protonation of the amine groups forms ammonium cations (–NH_3_^+^).

PoPD can be synthesized across a broad pH range, and the resulting polymer structure and conductivity depend strongly on the polymerization conditions [27,28]. When electropolymerized in acidic media (e.g., 0.1 M H_2_SO_4_ or buffers with pH ≈ 2–3), the polymer adopts a 1,4-benzoquinonediimine configuration, which exhibits higher electrical conductivity [29]. In contrast, polymerization under less acidic conditions (pH 4–6) favors the formation of a phenazine-type structure, which is electrically insulating (Figure 3) [30]. Because e-MIPs are most often synthesized in buffer solutions within a 4–6 pH range, the resulting PoPD films are typically non-conductive [31,32].

Like other polymers, PoPD can undergo doping through interaction with various chemical species, including HCl, Cu^2+^, H_3_BO_3_, Fe^3+^, and I_2_ [33]. The degree of doping and its effect on conductivity depend on the ratio of quinone- to phenazine-type segments in the polymer, as these determine the availability of nitrogen sites along the backbone for charge compensation [30,34]. Therefore, doping can modulate the charge-transfer properties of PoPD and, consequently, the electrochemical response of PoPD-based e-MIPs. To minimize these variations, it is advisable to maintain a consistent supporting electrolyte throughout experiments and to avoid prolonged exposure to highly saline or strongly acidic environments.

Overoxidation of PoPD may also occur under harsh electrochemical conditions, typically in 0.5 M H_2_SO_4_ at potentials between 0.8 and 0.9 V versus the Ag/AgCl reference electrode [35], leading to degradation of the polymer backbone and loss of redox activity. Despite these challenges, PoPD remains a highly stable polymer, outperforming polyaniline in thermal and chemical resistance. It can withstand temperatures up to approximately 312 °C and exhibit excellent corrosion resistance [36] features that contribute to its robustness and long-term durability in sensor applications.

## 3. Template Removal Strategies

**Figure 4:**
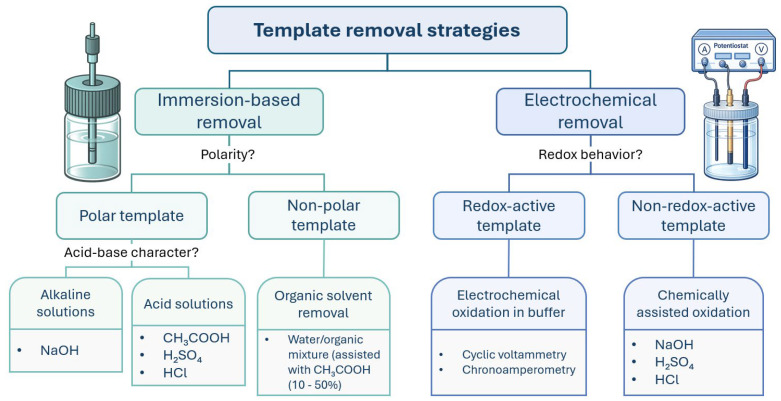
Template removal procedures in e-MIPs.

As illustrated in Figure 4, two main strategies are commonly used for template removal in e-MIPs: electro-cleaning and immersion. Among them, immersion is the simplest, non-instrumental method; however, it is often time-consuming, regardless of whether stirring is applied [37].

Electrocleaning consists of applying several potential sweeps on the MIP, typically through cyclic voltammetry within a defined potential window, with the purpose of inducing the oxidation or reduction of the embedded template molecules [38]. However, as noted earlier, the selected potential window that facilitates template desorption can also influence the oxidation state of the polymer matrix.

In contrast, immersion-based removal relies on the ability of the solvent to compete with the binding of the imprinted cavities and solvate the template, while minimizing damage to the polymer network [39]. As previously mentioned, this method often requires long incubation times, and such exposure has been associated with polymer swelling, unintended doping, and partial polymer leaching. For this reason, the structural and electrochemical quality of the polymer film should be routinely monitored to ensure that the removal procedure does not compromise the functional performance of the e-MIP.

### 3.1. Template Removal for Pyrrole-Based e-MIPs

In PPy-based e-MIPs, treatment with acidic or basic solutions promotes protonation or deprotonation of the polymer, respectively, inducing structural and charge rearrangements that facilitate the release of entrapped analytes [40]. For acidic treatments, weak protic acids are most commonly employed (Table 1), with acetic acid (CH_3_COOH) being the predominant choice, typically dissolved in water or organic solvents such as methanol [3,41,42,43]. This type of protic medium enables template removal while preserving the morphological integrity of the MIPs [41]. This can be considered a relatively mild removal medium, as its weak acidity minimizes the risk of damaging the polymer backbone or inducing significant corrosion. However, its effectiveness is highly dependent on the chemical nature of the target. As summarized in Table 1, this approach has been applied to targets such as lactate, glyphosate, and cortisol. In the case of lactate (pKa ≈ 3.9) and glyphosate, which contains multiple ionizable functional groups including amines, acetic acid promotes protonation, thereby facilitating disruption of hydrogen bonding, ultimately enabling template release. In contrast, cortisol does not undergo significant protonation under these conditions. Consequently, template removal relies primarily on weakening functional groups within the polymer cavity rather than modifying the target itself. This might result in incomplete removal, potentially leading to reduced binding capacity and selectivity in the resulting e-MIP.

In contrast, strong acids such as HCl have also been used, like in the case of Kanamycin as shown in Table 1, to promote a stronger protonation of the target. These conditions can also protonate the PPy backbone [40], therefore, inducing substantial polymer swelling, a phenomenon likely linked to changes in the protonation state of the PPy backbone. Such perturbations can progressively disrupt the polymer structure, ultimately leading to partial denaturation [15,40,44]. Nevertheless, immersion in strong acidic media has been less frequently reported in the literature, and short times are employed.

On the other hand, for alkaline treatments, NaOH solutions around 0.1 M are most commonly employed for template removal [15,38,45]. At these moderate concentrations, NaOH promotes a milder deprotonation of PPy; under these conditions, targets containing ionizable functional groups can undergo deprotonation, facilitating their removal from the imprinted cavities. For instance, thiol-containing molecules such as ethanethiol readily deprotonate to form thiolate anions, while salicylic acid is converted to its corresponding carboxylate form. These transformations increase solubility and weaken hydrogen bonding interactions, thereby promoting efficient removal.

In contrast, other targets such as dimethoate and lactose may undergo chemical transformations in alkaline media. Dimethoate is susceptible to base-catalyzed hydrolysis, leading to degradation of the parent molecule, whereas lactose, as a reducing sugar, can become more reactive through ring opening and enediol formation. These processes can facilitate template removal; however, they may also alter the chemical identity of the template, potentially affecting the fidelity of the imprinted cavities.

Additionally, strongly alkaline conditions can be detrimental to PPy stability. According to Jin et al. (2019) [46], PPy films immersed in 0.5 M NaOH exhibit progressive degradation, including increased internal resistance, reduced electrochemical activity, and structural damage to the conjugated polymer backbone. The authors attribute this deterioration primarily to OH^−^ attack, which is further intensified in aerated media by electrochemical degradation processes [46,47,48].

To modulate the solubility of the target or its transformation products, acids and bases are often combined with organic or aqueous solvents such as ethanol, methanol, or water [3,41,45,49]. This approach allows fine control over solvation properties and enhances template removal efficiency. In those cases, immersion remains the simplest and most versatile method for template removal in e-MIPs, as the wide range of available solvent systems, together with tunable pH conditions, enables multiple pathways for efficient target removal.

Despite their simplicity, immersion-based protocols can vary significantly in duration, from only a few minutes [43,44,49,50] to more than one hour [42], depending not only on the strength of the template–polymer interactions but also on the thickness and density of the polymer film. These structural properties are governed by electropolymerization parameters, particularly the number of cyclic voltammetry (CV) cycles and the scan rate. As summarized in Table 1, most PPy-based e-MIPs are prepared using approximately 5–10 cycles and scan rates in the range of 25–100 mV s^−1^, conditions that directly influence film compactness and, consequently, template diffusion during template removal.

As a general guideline, maintaining low concentrations of acids or bases, and preferably employing weak acids, helps preserve polymer integrity. Concurrently, careful adjustment of pH can promote target dissociation by leveraging intrinsic chemical properties such as pKa-dependent protonation equilibria or base-catalyzed transformations, thereby facilitating template removal.

Another strategy for template removal relies on electrocleaning (Table 1), where many other authors assure that it provides a faster and more efficient alternative for template removal while better preserving the structural and chemical integrity of the polymer matrix [37,47,51]. This approach relies on electrochemical techniques, such as CV, to induce template release through the application of controlled potentials. In the case of CV, the potential window can be adjusted according to the polymer stability. Mild potentials, ranging from −0.1 to 0.9 V, generally require a higher number of cycles (around 10) to achieve effective template removal [52,53]. In contrast, strongly oxidizing potentials, extending up to 1.6 V, can promote template removal with significantly fewer cycles (around 5–7) [54,55,56].

Additionally at high anodic potential, another phenomenon arises: polymer overoxidation. This reaction occurs when pyrrole units are driven beyond their conductive state and become susceptible to nucleophilic attack, most prominently by hydroxide ions. Importantly, this process does not proceed under neutral or acidic conditions but is favored in alkaline environments where strong nucleophiles are abundant. Overoxidation leads to structural modification of the polymer, including the formation of oxygen-containing groups such as carbonyl and carboxyl functionalities, accompanied by a marked reduction in electrical conductivity. When carefully controlled, this can facilitate the release of entrapped template molecules, thereby improving removal efficiency before subsequent chemical removal. However, excessive overoxidation can disrupt the integrity of the polymer network, promote delamination, and ultimately compromise the electrochemical readout of the e-MIP. Overall, while overoxidation can assist template removal under alkaline conditions, its impact on network stability and conductivity necessitates careful optimization of the potential window and removal medium.

Another strategy reported in the literature is applying a two-step removal template strategy. According to Zvirzdine et al. (2025), combining electrocleaning under mild conditions followed by immersion in a basic solution removes efficiently and preserves polymer integrity [57]. The initial step gently disrupts polymer-template interactions, removing loosely bound species without compromising the e-MIP surface. The following step facilitates deprotonation and opens molecular cavities, ensuring thorough removal of strongly bound templates [57]. These findings indicate that the conditions required for successful template release are strongly dependent on the chemical stability and crosslinking density of the polymer.

As shown in Table 1, electrocleaning via CV can be performed in a variety of media, including acidic, alkaline, and buffered solutions. In this approach, the applied potential serves as the primary driving force for disrupting template–polymer interactions and promoting template release. While many of the considerations discussed for immersion-based removal remain applicable, electrocleaning introduces additional parameters associated with the CV protocol.

Once an appropriate potential window has been established, the scan rate must be carefully optimized. This parameter is closely related to the internal structure of the polymer, particularly its porosity, as the diffusion of the template out of the network depends on the effective time under applied potential. Slower scan rates can enhance diffusion-driven removal, whereas faster scans may limit template release efficiency.

A key advantage of this electrocleaning protocol is the ability to monitor the current response in real time, providing insight into both template removal and polymer integrity. A progressive decrease in current may indicate loss of conductivity, often associated with polymer degradation or overoxidation, whereas an increase in current can reflect redox activity of the released template or the presence of electroactive species at the electrode surface.

**Table 1:** Reported template removal procedures for pyrrole-based MIPs.

Monomer	Target	Electrode	Synthesis Conditions	Removal Method	Removal Conditions	Removal Solution	Reference
Pyrrole	Cortisol	SPCE/PB/NiHCF	CV [−0.2 to 0.9] V 10 cycles 25 mV s^−1^	Immersion	10 min	CH_3_COOH 8%	[43]
Pyrrole	Lactate	SPCE/PB	CV [−0.2 to 0.9] V 10 cycles 25 mV s^−1^	Immersion	120 min	CH_3_COOH 8%	[42]
Pyrrole	Cortisol	LIG	CV [−0.2 to 0.9] V 10 cycles 50 mV s^−1^	Immersion	30 min	CH_3_COOH/MetOH (7:3 *v*/*v*)	[3]
Pyrrole	Glyphosate	AuE	Chronoamperometry 1.5 V 5 s	Immersion	30 min	CH_3_COOH/MetOH (1:1 *v*/*v*)	[41]
Pyrrole	Melamine	GCE/ERGO	CV [−0.4 to 1] V 5 cycles	1—Immersion 2—Immersion	1—5 min 2—5 min	1—H_2_O_2_–NaOH (0.1 M) in a mixed ACN-H_2_O 2—ABS (pH 5.2)	[50]
Pyrrole	Rifampicin and Isoniazid	GCE/Cu-MOF/MC	CV [−0.5 to 0.8] V 10 cycles 50 mV s^−1^	Immersion under stirring	15 min	MetOH/H_2_O (1:1, *v*/*v*)	[49]
Pyrrole	Kanamycin	GCE/GO	CV [0 to 1.8] V 5 cycles 50 mV s^−1^	Immersion under stirring	10 min	HCl (0.01 M)	[44]
Pyrrole	Ethanethiol	GCE	CV [−0.6 to 1.8] V 8 cycles 50 mV s^−1^	Immersion under stirring	30 min	NaOH (0.2 M):EtOH (8:2, *v*/*v*)	[45]
Pyrrole	Dimethoate	AuE	CV [−0.4 to 1.5] V 10 cycles 50 mV s^−1^	Immersion under stirring	45 min	NaOH (0.1 M)	[15]
Polypyrrole/1-decanesulfonate	Lactose	AuE	Chronoamperometry 1.2 V 300 s	Immersion under stirring	30 min	NaOH (0.1 M)	[38]
Pyrrole	Cortisol	SPCE	CV [−0.2 to 0.9] V 15 cycles 50 mV s^−1^	Electrocleaning	CV [−0.2 to 0.9] V 10 cycles	PBS	[52]
Pyrrole/anionic β cyclodextrin	Dopamine	GCE	Potentiostatic electropolimerization 0.80 V 0.32 C cm^−2^	Electrocleaning	CV [−0.1 to 0.9] V vs SCE 10 cycles 100 mV s^−1^	Na_2_SO_4_ (0.1 M)	[53]
Pyrrole-phenylboronic	Dopamine	GCE	CV [−0.2 to 1.2] V 20 cycles 50 mV s^−1^	Electrocleaning	CV [0 to 1.5] V 6 cycles	H_2_SO_4_ (0.5 M)	[54]
Pyrrole	Cortisol	GCE	CV [−1 to 1] V 10 cycles 50 mV s^−1^	Electrocleaning	CV (potential window not specified) 20 cycles 50 mV/s	PBS	[58]
Pyrrole-histidine	Teriflunomide	GCE	CV [−0.2 to 1.6] V 10 cycles 100 mV s^−1^	Electrocleaning	CV (potential window and scan rate not specified) 7 cycles	NaCl (0.25 M) in PBS (pH 7.4) 1:1	[56]
Pyrrole	Lactose	PE	CV [0.0 to 1.2] V 5 cycles 50 mV s^−1^	Electrocleaning	CV [0 to 1.6] V 5 cycles 50 mV s^−1^	NaOH 0.1 M	[55]
Pyrrole	Insulin	SPCE	CV [0.0 to 0.9] V 10 cycles 50 mV s^−1^	Electrocleaning	CV [−0.2 to 1.0] V 25 cycles 50 mV s^−1^	PBS (0.01 M, pH 7.2)	[47]
Pyrrole	Salicylic Acid	Platinum electrode	Amperometric electropolimerization 0.80 V 60 s	1—Electrocleaning 2—Immersion under stirring	1—CV [0 to 0.6] V 50 cycles 50 mV s^−1^ 2—15 min–200 rpm	1—NaOH (0.1 M) 2—NaOH (0.1 M)	[57]

SPCE/PB/NiHCF: Screen-printed carbon electrodes modified with Prussian Blue nanoparticles and stabilized with nickel hexacyanoferrate. AuE: Gold electrode. GCE: Glassy carbon electrode. GCE/GO: GCE coated with graphene oxide. LIG: Laser-induced graphene. GCE/ERGO: GCE modified with electrochemically reduced graphene oxide. ACN: Acetonitrile. GCE/Cu-MOF/MC: GCE modified with copper metal–organic framework/mesoporous carbon. PBS: Phosphate-buffered solution. SCE: Saturated calomel electrode. PE: Graphite paper electrode.

### 3.2. Template Removal for Ortho-Phenylamine-Based e-MIPs

Among the template removal strategies outlined in Figure 4, simple immersion remains the most widely adopted approach for oPD-based e-MIPs (Table 2). Both acidic and alkaline media have been used effectively; in PoPD e-MIPs, prepared from 10 to 30 CV cycles and scan rates from 50 to 200 mV s^−1^, these conditions require times from 5 min to prolonged incubation periods like overnight to achieve complete template removal [59,60,61].

Notably, template removal can be reduced to only a few minutes by selecting a more suitable solvent, which promotes rapid disruption of monomer–target intermolecular interactions and accelerates diffusion from the polymer matrix [9,62]. For example, Waffo et al. (2018) reported an o-PD-based e-MIP for artemisinin detection prepared using 10 electropolymerization cycles; however, template removal still required more than 2 h in 0.1 M NaOH [61]. Conversely, Liu et al. fabricated an o-PD-based e-MIP for triclosan using 20 electropolymerization cycles and the same scan rate and potential window as those reported by Waffo et al. [61] but achieved complete template removal in only 10 min [63]. Although multiple variables could contribute to this contrast, the most plausible explanation arises from the distinct alkaline behavior of the two template molecules. Triclosan undergoes deprotonation of its phenolic group in NaOH, generating a phenolate species with greatly enhanced aqueous solubility, thus enabling rapid diffusion out of the polymer. In contrast, artemisinin does not ionize under these conditions and displays low diffusivity in basic media, as it preferentially dissolves in polar organic solvents [64]. Overall, the comparison exemplifies how the physicochemical properties of the template and its interaction with the removal solvent could influence the kinetics of template removal.

Immersion under stirring is also used as a template removal strategy to enhance mass transfer between the eluent and the polymer surface. The literature consistently relies on binary solvent systems whose composition is selected according to the solubility profile of the target analyte and the type of intermolecular interactions governing template entrapment. Mostly using combinations of acetic acid [65,66,67] or alcohols like methanol or ethanol [10,17]. An alternative configuration consists of immersion under flow-injection operation, where template removal occurs in a continuous stream of solvent rather than a static solvent bath. This strategy enhances convective mass transfer and reduces the likelihood of re-adsorption [68]. However, the increased solvent consumption associated with this strategy may limit its applicability from both economic and sustainability perspectives.

Another observation in the template removal step is the wide diversity of reported protocols, many of which do not appear to follow a clear selection criterion. For example, removing dopamine from an e-MIP using concentrated H_2_SO_4_ for 5 h appears excessively harsh, given both the highly corrosive nature of the acid and the risk of introducing sulfonated moieties or other chemical alterations within the polymer network [59].

Other examples include e-MIPs for artemisinin, resorcinol, and triclosan, all washed using 0.1 M NaOH, a mildly alkaline medium. In the case of artemisinin, the molecule would not significantly ionize under these conditions, so its diffusion out of the polymer network would not be strongly favored by the removal solution itself [61]. In contrast, for resorcinol and triclosan, with pKa values around 9 and 8, respectively, NaOH would indeed promote deprotonation, increasing their solubility and facilitating their removal from the polymer [63,69].

Among the reported removal solutions, those containing CH_3_COOH seem to provide some of the shortest removal times, typically spanning 10 to 20 min. For propachlor, ecstasy, chlorpyrifos, and sucrose, CH_3_COOH-containing media have all been reported for removal [65,66,67]. However, from a chemical standpoint, only ecstasy would be expected to undergo significant protonation under these conditions, thereby becoming more soluble in the acidic medium [27]. In the other cases, the role of acetic acid may be primarily to weaken interactions involving the functional groups within the polymer cavity, while the co-solvent likely contributes more directly to target release through improved solubility.

In contrast to PPy-based e-MIPs, where electrocleaning is commonly employed to accelerate template removal via controlled overoxidation, this strategy is less frequently used in o-PD systems. The lower use of electrocleaning is generally associated with the higher structural sensitivity of PoPD, whose physicochemical properties are strongly influenced by hydrogen bonding, conjugation, and molecular strain [62]. Under harsh electrochemical conditions, the film may therefore be modified along with the template-elution process. Accordingly, only a limited number of studies have reported electrocleaning in o-PD-based e-MIPs [70,71,72]. In the cases where electrochemical template removal has been implemented, CV protocols have been used to facilitate template elution by promoting localized electrochemical perturbations at the polymer–solution interface.

In conclusion, immersion is a frequently favored strategy for template removal in this system, primarily because poly(o-PD) imprinted films are inherently much thinner than those from more conductive polymers like PPy. This limited film thickness arises from the low conductivity of poly(o-PD), which passivates the electrode and leads to the formation of self-limiting layers typically below 100 nm [71]. Consequently, mass-transfer barriers are minimized, enabling solvents to efficiently penetrate and solvate trapped templates without aggressive electrochemical conditions. Thus, immersion-based removal offers a gentler approach that avoids overoxidation, structural damage, and loss of electroactivity, thereby preserving the integrity and performance of the e-MIP.

The mechanistic considerations summarized in Figure 4 are derived directly from the experimental trends discussed throughout this review. In particular, the choice of template removal strategy is primarily governed by three factors: the solubility of the target, its ionization behavior (pKa-dependent), and its redox activity. These parameters determine whether template removal is driven predominantly by chemical solvation, pH-induced dissociation, or electrochemical transformation. Accordingly, Figure 4 provides a comparative overview of how target properties and polymer chemistry influence the selection and effectiveness of different template removal approaches in e-MIPs.

**Table 2:** Reported template removal procedures for o-phenylenediamine-based MIPs.

Monomer	Target	Electrode	Synthesis Conditions	Removal Method	Removal Conditions	Removal Solution	Reference
*o*-pd	Dopamine	AuE	CV [0 to 0.8] V 30 cycles 100 mV s^−1^	Immersion	5 h	H_2_SO_4_ (0.5 M)	[59]
*o*-PD*-co-o-AP*	Prostate-specific antigen	SPCE	CV [−0.2 to 1.2] V 10 cycles 50 mV s^−1^	Immersion	12 min	H_2_C_2_O_4_ (5 mM)	[9]
*o*-pd	Theophylline	AuNPs/GCE	CV [0 to 0.80] V 20 cycles 50 mV s^−1^	Immersion	5 h	EtOH	[60]
*o*-pd	Thymol	GCE	CV [0 to 0.8] V 10 cycles 50 mV s^−1^	Immersion	5 min	EOH:H_2_O (1:1 *v*/*v*)	[62]
*o*-pd	Artemisinin	Au wires	CV [0 to 0.8] V 10 cycles 50 mV s^−1^	Immersion	2.5 h	NaOH 0.1 M	[61]
*o*-pd	Resorcinol	GCE	CV [0 to 0.8] V 20 cycles 50 mV s^−1^	Immersion	Overnight	NaOH 0.1 M	[69]
*o*-pd	Triclosan	GCE	CV [0 to 0.8] V 20 cycles 50 mV s^−1^	Immersion	10 min	NaOH 0.1 M	[63]
*o*-PD-co-Py	Propachlor	GCE/ERGO	CV [0 to 1.1] V 15 cycles 100 mV s^−1^	Immersion	15 min	CH_3_COOH:MeOH (1:9 *v*/*v*)	[65]
*o*-pd	Ecstasy	SPCE	CV [0 to 1.2] V 5 cycles 100 mV s^−1^	Immersion under stirring	10 min	CH_3_COOH:H_2_O (1:1 *v*/*v*)	[27]
*o*-PD-co-MD	Chlorpyrifos	Au-NP@PG	CV [−0.1 to 0.9] V 8 cycles 100 mV s^−1^	Immersion under stirring	20 min	CH_3_COOH glacial: MeOH (8:2 *v*/*v*)	[66]
*o*-pd	Sucrose	GCE/MWCNT	CV [−0.4 to 1] V 25 cycles 50 mV s^−1^	Immersion under stirring	12 min	CH_3_COOH:ACN (1:5 *v*/*v*)	[67]
*o*-pd	2,4-D	Au-SPE	CV [0 to 1] V 10 cycles 200 mV s^−1^	Immersion under stirring	15 min	MeOH:H_2_O (70:30)	[17]
*o*-pd	2,4-D	Au disk electrode	CV [0 to 1] V 10 cycles 200 mV s^−1^	Immersion under stirring	15 min	MeOH:H_2_O (1:1 *v*/*v*)	[10]
*o*-pd and aniline	Met-enkephalin	CFM	CV [−0.1 to 1.25] V 20 cycles 50 mV s^−1^	Immersion under flow injection	108.4 mL h^−1^ 10 min	PBS	[68]
*o*-pd	Entacapone	GCE	CV [0 to 0.8] V 10 cycles	Electrocleaning	CV [0 to 0.8] V	ABS (0.2 M, pH 4.5)	[70]
*o*-pd	Cortisol	SPCE	CV [0 to 1] V 30 cycles 50 mV s^−1^	Electrocleaning	CV [−0.2 to 0.8] V 25 cycles 50 mV s^−1^	PBS (0.01 M)	[71]
*o*-pd	Serotonin and Glutamate	GCE/NRGO	CV [0 to 0.8] V 15 cycles 50 mV s^−1^	Electrocleaning	Potentiostatic 400 s	HCl 0.5 M	[72]

*o*-pd: *o*-phenylenediamine, 2,4-D: 2,4-dichlorophenoxyacetic acid. Au-SPE: Screen-printed gold electrodes. GCE: Glassy carbon electrode. GCE/MWCNT: GCE modified with multiwall carbon nanotubes. CFM: Carbon fiber microelectrode. ABS: Acetate buffer solution. SPCE: Screen-printed carbon electrode. PBS: Phosphate-buffered solution. *o*-PD-co-Py: *o*-phenylene diamine-co-pyrrole. GCE/ERGO: GCE modified with electrochemically reduced graphene oxide. *o*-PD-*co-o-AP*: o-pd copolymerized with *o*-aminophenol. *o*-PD-co-MD: o-pd copolymerized with methyldopa. Au-NP@PG: gold nanoparticles on pencil graphite.

## 4. Challenges and Future Directions

One main challenge in reproducing e-MIPs that limits their practical application is the lack of specific details or clarity in the production protocols of e-MIPs [2,7,73,74,75,76,77,78,79,80]. The reproducibility of e-MIPs is frequently compromised by inconsistencies in the manufacturing steps, especially in the electropolymerization conditions, the removal of the template molecule, etc. In many cases, there is no detailed information on the need to evaluate the interaction time between the template molecule and the functional monomer for the formation of an intermediate complex before electropolymerization. This step is fundamental to promoting a more efficient interaction between the template molecule and the monomer and can take from minutes to hours, ultimately resulting in more selective cavities. Another aspect frequently poorly described in the literature is the analyte rebinding process to the binding sites [81,82]. Several protocols omit or do not adequately specify the conditions necessary for efficient rebinding, which compromises the reproducibility of the results. Additionally, the absence or limited use of non-imprinted sensors (NIPs) in optimization steps, often due to time constraints, resource limitations, or manufacturing complexity, can lead to misinterpretations of data and a lack of standardization in e-MIP development protocols.

These variations can result in differences in the quality of the printed cavities and, consequently, in the sensor’s performance [2,7,80]. Furthermore, difficulties associated with the renewal and immobilization of e-MIPs on electrode surfaces also contribute to it. This problem is exacerbated using conventional electrodes, which may exhibit mechanical fragility, such as crack formation and delamination [73,74].

One of the fundamental steps is template removal, as it directly affects the sensor’s selectivity. When attempting to reproduce protocols reported in the literature, it becomes evident that many studies do not adequately describe or evaluate the template removal protocol, particularly when using solvents with strong acids for template removal, even when the chemical nature of polymer and target would not require these conditions, making reproducibility difficult. Inefficient removal or polymer damage can lead to non-specific interactions and reduced sensor selectivity, finally compromising reproducibility [2,75].

Advancing e-MIPs will require a shift toward a more systematic and mechanistic understanding of the template-removal step, which remains one of the least studied yet structurally impactful stages of MIP fabrication. As highlighted in this review, removal conditions, whether acidic, alkaline, organic, or electrochemical, can significantly modify polymer oxidation states, dopant composition, porosity, and mechanical stability, ultimately altering the fidelity of the imprinted cavities and the electrochemical response of the sensor. Despite this, template removal procedures still rely largely on empirical choices, while systematic studies evaluating key parameters such as pH, solvent composition, extraction time, applied potential, and polymer stability remain scarce. Consequently, future efforts should therefore incorporate in situ analytical techniques and advanced surface characterization to directly correlate template removal chemistry with polymer restructuring dynamics. Computational modeling and monomer–template interaction, which have previously been applied to bulk MIPs for rational monomer selection and optimization of polymerization conditions [83], could be further extended to the selection of template removal conditions in e-MIPs. Promising hybrid strategies, such as controlled partial overoxidation, mild multi-step electrochemical cleaning, or the incorporation of conductive nanomaterials to stabilize films during aggressive washes, warrant further exploration. Establishing standardized, reproducible template removal guidelines across laboratories will be essential to improving sensor robustness and comparability. Ultimately, a deeper understanding of how template removal conditions reshape the polymer network will be key to designing next-generation e-MIPs with higher selectivity, enhanced stability, and reliable performance in real-world applications.

## Data Availability

No new data was created.

## Abbreviations

The following abbreviations are used in this manuscript:

e-MIP	Electropolymerized Molecularly Imprinted Polymer
MIP	Molecularly Imprinted Polymer
NIP	Non-Imprinted Polymer
PPy	Polypyrrole
Py	Pyrrole
PoPD	Poly(o-phenylenediamine)
oPD	o-Phenylenediamine
PFAS	Per- and Polyfluoroalkyl Substances
HPLC	High-Performance Liquid Chromatography
UV/Vis	Ultraviolet/Visible Spectroscopy
HNMR	Proton Nuclear Magnetic Resonance
CV	Cyclic Voltammetry
PBS	Phosphate-Buffered Solution
SPCE	Screen-Printed Carbon Electrode
AuE	Gold Electrode
GCE	Glassy Carbon Electrode
